# Expression of granzyme B sensitizes ALK+ ALCL tumour cells to apoptosis-inducing drugs

**DOI:** 10.1186/1476-4598-13-199

**Published:** 2014-08-29

**Authors:** Joel D Pearson, Jingxi Zhang, Zuoqiao Wu, Kayla D Thew, Katelynn J Rowe, Julinor TC Bacani, Robert J Ingham

**Affiliations:** Department of Medical Microbiology and Immunology and Li Ka Shing Institute of Virology, University of Alberta, Katz Group Centre for Pharmacy and Health Research, University of Alberta, Edmonton, AB T6G 2E1 Canada; Department of Laboratory Medicine and Pathology, University of Alberta, Edmonton, AB T6G 2B7 Canada

**Keywords:** Granzyme B, Lymphoma, ALK+ ALCL, Apoptosis

## Abstract

**Background:**

The serine protease Granzyme B (GzB) is primarily expressed by cytotoxic T lymphocytes and natural killer cells, and functions in allowing these cells to induce apoptosis in virally-infected or transformed cells. Cancers of both lymphoid and non-lymphoid origin also express GzB, and in some cases this expression has been linked to pathogenesis or sensitizing tumour cells to cell death. For example, GzB expression in urothelial carcinoma was implicated in promoting tumour cell invasion, whereas its expression in nasal-type NK/T lymphomas was found to correlate with increased apoptosis. GzB expression is also a hallmark of the non-Hodgkin lymphoma, anaplastic lymphoma kinase-positive, anaplastic large cell lymphoma (ALK+ ALCL). Given the fact that ALK+ ALCL exhibits high levels of apoptosis and is typically responsive to conventional chemotherapy, we examined whether GzB expression might play a role in sensitizing ALK+ ALCL tumour cells to apoptosis.

**Methods:**

ALK+ ALCL cell lines stably expressing GzB or non-targeting (control) shRNA were generated and apoptosis was examined by anti-PARP western blotting and terminal deoxynucleotidyl transferase dUTP nick end labelling. Both spontaneous apoptosis and apoptosis in response to treatment with staurosporine or doxorubicin were investigated. In order to assess whether additional granzymes might be important in promoting cell death in ALK+ ALCL, we examined whether other human granzymes were expressed in ALK+ ALCL cell lines using reverse-transcriptase PCR and western blotting.

**Results:**

Expression of several GzB shRNAs in multiple ALK+ ALCL cell lines resulted in a significant decrease in GzB levels and activity. While spontaneous apoptosis was similar in ALK+ ALCL cell lines expressing either GzB or control shRNA, GzB shRNA-expressing cells were less sensitive to staurosporine or doxorubicin-induced apoptosis as evidenced by reduced PARP cleavage and decreased DNA fragmentation. Furthermore, we found that GzB is the only granzyme that is expressed at significant levels in ALK+ ALCL cell lines.

**Conclusions:**

Our findings are the first to demonstrate that GzB expression sensitizes ALK+ ALCL cell lines to drug-induced apoptosis. This suggests that GzB expression may be a factor contributing to the favourable response of this lymphoma to treatment.

## Background

Granzyme B (GzB) is a serine protease found in the cytoplasmic granules of cytotoxic T lymphocytes (CTLs) and natural killer (NK) cells. It is released along with other granule proteins such as the pore-forming protein perforin, into the immunological synapse where it is translocated into virally-infected or transformed cells to initiate apoptosis [1, 2]. GzB primarily initiates apoptosis either through direct proteolytic processing and activation of initiator (caspase 8) or executioner (caspases 3 and 7) caspases, or through disruption of mitochondrial outer membrane potential via cleavage of the Bid protein [2]. GzB may also contribute to apoptosis induction through the cleavage of other cellular substrates including nuclear lamin B, poly (ADP-ribose) polymerase (PARP), and inhibitor of caspase-activated DNase (ICAD) [2]. GzB also has substrates outside the cell, and may gain access to the extracellular space as a result of leakage from the immunological synapse or by direct secretion from cytotoxic cells [3, 4]. These substrates include extracellular matrix (ECM) proteins such as vitronectin, laminin, fibronectin, decorin, and biglycan [5–7]. Cleavage of these proteins by GzB has been implicated in detaching adherent cells bound to these proteins [5], promoting the migration of cells through matrigel [6], and releasing matrix-bound cytokines [7].

Not surprisingly, GzB is expressed in a number of T/NK cell neoplasms including anaplastic lymphoma kinase-positive, anaplastic large cell lymphoma (ALK+ ALCL) [8–11], ALK- ALCL [8–10], and nasal-type NK/T cell lymphoma [10, 12], as well as other lymphoid cancers including some Hodgkin lymphoma [13–15] and multiple myeloma [16]. GzB expression can also be found in cell lines and/or primary tumours of non-lymphoid origin including acute myeloid leukemia [17], prostate cancer [18] and some carcinomas of the breast [19] and urothelium [6]. While the significance, if any, of GzB expression in most of these malignancies has yet to be firmly established, in urothelial carcinoma GzB expression correlates with increased invasiveness and epithelial-to-mesenchymal transition of tumour cells [6]. More importantly, this study demonstrated that in GzB-expressing bladder cancer cell lines, GzB promotes the invasion of these cells through matrigel, presumably through the degradation of ECM proteins within the matrigel [6]. In contrast, GzB expression in some cancers has been reported to be detrimental to tumour cells. In nasal-type NK/T cell lymphomas, GzB expression correlates with increased apoptosis, and leakage of GzB out of cytotoxic vesicles in the HANK-1 NK/T cell lymphoma cell line was argued to contribute to high levels of spontaneous apoptosis in this cell line [12]. Similarly, prostate cancer cell lines treated with resveratrol and radiation up-regulate GzB and perforin, and increased expression of these proteins was postulated to contribute to tumour cell apoptosis [18].

ALK+ ALCL is an aggressive non-Hodgkin T cell lymphoma that is most prevalent in children and young adults [20]. This lymphoma expresses GzB in cytotoxic granules along with perforin and T cell-restricted intracellular antigen-1 (TIA-1) [8–11]. Lymphoma cells typically possess T cell receptor gene rearrangements [8, 11, 21]; however, the expression of many T cell markers is variable in ALK+ ALCL [11, 21, 22]. A defining feature of ALK+ ALCL is chromosomal translocations or inversions involving the *ALK* tyrosine kinase gene [23, 24]. These chromosomal alterations generate oncogenic fusion proteins, the most common being NPM-ALK. NPM-ALK initiates a number of down-stream signalling events that ultimately promote the proliferation, survival, and migration of ALK+ ALCL tumour cells [25, 26]. In previous work, we demonstrated that *GzB* transcription is promoted by NPM-ALK signalling in ALK+ ALCL, largely through the AP-1 family transcription factor, JunB [27].

Given that ALK+ ALCL tumour cells exhibit high levels of apoptosis [28–30] and the observed correlation between GzB expression and apoptosis rate in nasal-type NK/T lymphomas [12] and prostate cancer cell lines [18], we decided to investigate whether GzB expression might sensitize ALK+ ALCL cells to apoptosis. We demonstrate that short-hairpin RNA (shRNA)-mediated knock-down of GzB in ALK+ ALCL cell lines is associated with decreased GzB enzymatic activity. Furthermore, we show that while knock-down of GzB does not influence spontaneous apoptosis in ALK+ ALCL cell lines, it reduces drug-induced apoptosis in these cells. GzB is one of five human granzymes, and all of these proteins have been implicated in promoting programmed cell death. Therefore, we examined whether other granzymes were expressed in these cell lines and found that GzB is the only human granzyme expressed at significant levels. In sum, our findings demonstrate that a well-known phenotypic characteristic of ALK+ ALCL may be an important factor underlying the ability to treat this lymphoma.

## Results

### GzB protein levels and activity are significantly reduced in ALK+ ALCL cell lines treated with GzB shRNA

**Figure 1:**
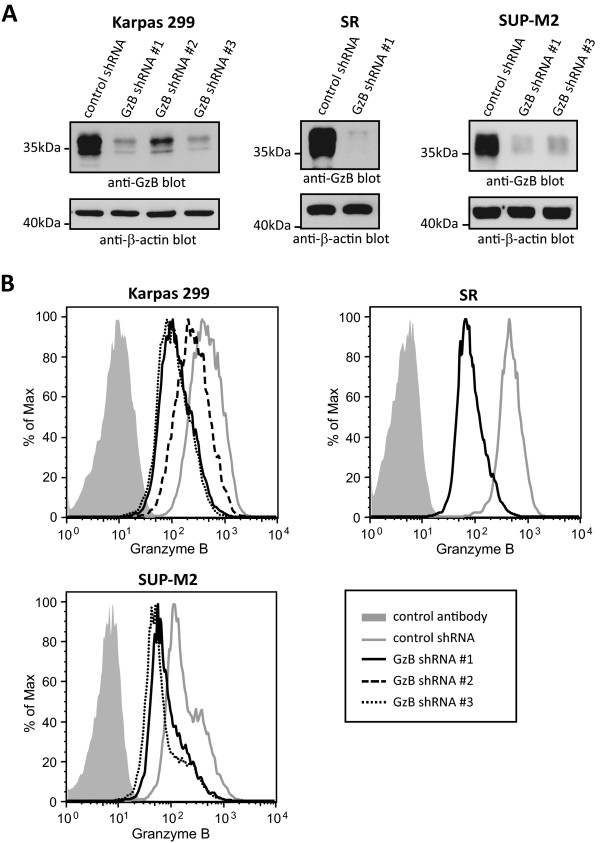
**Knock-down of GzB in ALK+ ALCL cell lines.** Western blots **(A)** or flow cytometry plots **(B)** comparing the expression of GzB in ALK+ ALCL cell lines stably expressing either a non-targeting (control) shRNA or the indicated GzB shRNAs. Molecular mass standards are indicated to the left of the western blots.

**Figure 2:**
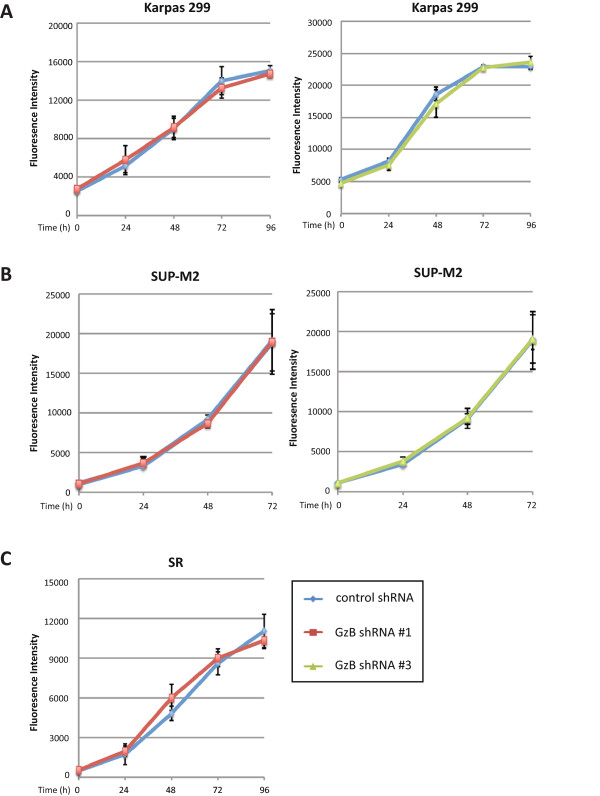
**Knock-down of GzB in ALK+ ALCL cell lines does not affect growth rate.** The growth rate of Karpas 299 **(A)**, SUP-M2 **(B)**, or SR **(C)** cells expressing the indicated GzB shRNA was compared to control shRNA-expressing cells. The results represent the average and standard deviation of 3 (SUP-M2) or 4 (Karpas 299 and SR) independent experiments. The only time point where we observed a statistically significant difference between the GzB and control shRNA-expressing cell lines was at the 48 h time point where the GzB shRNA #1-expressing SR cell line was modestly higher than the control shRNA-expressing cells (*p* = 0.042).

In order to examine whether GzB sensitizes ALK+ ALCL to apoptosis, we generated ALK+ ALCL cell lines where GzB expression had been stably knocked-down with shRNA. We generated these knock-down cells in multiple ALK+ ALCL cell lines (Karpas 299, SUP-M2, and SR (also known as SR-786)) and used shRNAs that target different regions of the *GzB* gene. Analysis of GzB knock-down by western blotting (Figure 1A) or flow cytometry (Figure 1B) demonstrated that GzB protein levels were significantly reduced in cells expressing GzB shRNAs compared to cells expressing a non-targeting control shRNA. Quantification of the mean fluorescence intensity of GzB staining indicated that GzB protein levels in the GzB knock-down cells were 18 to 49% of the levels in cells expressing the non-targeting shRNA (Table 1). Of note, GzB knock-down cell lines had a similar growth rate as cells expressing control shRNA (Figure 2).

**Table 1:** **Quantification of GzB levels in ALK+ ALCL cell lines expressing GzB shRNA**

Cell line	shRNA construct	Relative GzB expression (% of control shRNA)
Karpas 299	GzB shRNA #1	28.6 +/− 3.2
	GzB shRNA #2	48.9 +/− 7.8
	GzB shRNA #3	23.8 +/− 1.4
SR	GzB shRNA #1	18.5 +/− 2.8
SUP-M2	GzB shRNA #1	37.6 +/− 7.5
	GzB shRNA #3	35.1 +/− 2.9

The relative mean fluorescence intensity of GzB staining in Karpas 299, SR, or SUP-M2 cells stably expressing the indicated GzB shRNA constructs compared to cells expressing control, non-targeting shRNA is indicated. The values shown represent the average and standard deviation of 3 independent experiments.

**Figure 3:**
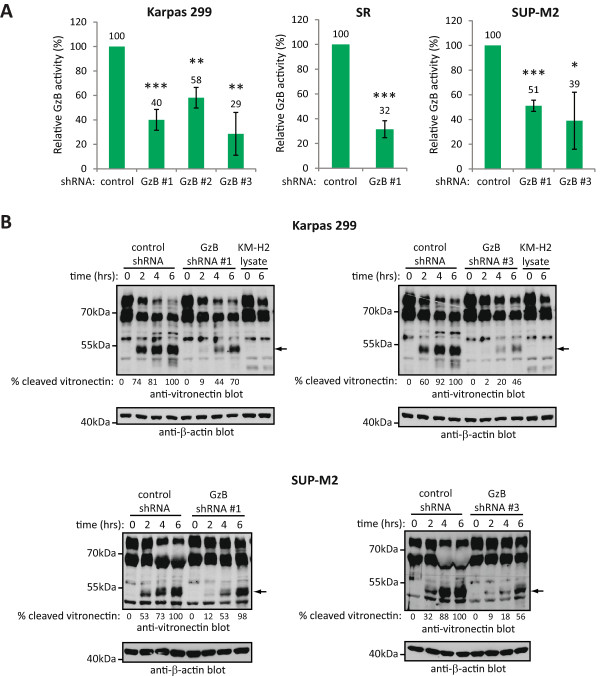
**ALK+ ALCL cell lines with GzB knock-down show significant reduction in GzB activity. A**. Cleavage of the synthetic GzB substrate, Ac-IEPD-pNA, was examined using lysates from the indicated ALK+ ALCL cell lines expressing either control or GzB shRNA. The results represent the average and standard deviation of 3 independent experiments. *p* values comparing cells expressing GzB shRNA to cells expressing control shRNA were obtained by performing paired, one-tailed t-tests. *p < 0.05, **p < 0.01, ***p < 0.005. **B**. Lysates from Karpas 299 cells (above) or SUP-M2 cells (below) expressing the indicated shRNAs were incubated with purified vitronectin for the indicated times and vitronectin cleavage was examined by western blotting. Arrows indicate the major observed vitronectin cleavage product. Cleavage of vitronectin was also examined when incubated with lysates from the GzB-negative, Hodgkin lymphoma cell line, KM-H2, which served as a negative control. Anti-β-actin blots were performed to demonstrate that equivalent amounts of lysate were added to the reactions. The percentage of cleaved vitronectin (% cleaved vitronectin) was determined by densitometry, and represents the percentage of cleaved vitronectin in each lane relative to cleaved vitronectin in the lane where vitronectin was incubated with control shRNA lysate for 6 h. Molecular mass standards are indicated to the left of the western blots.

GzB knock-down resulted in markedly reduced GzB enzymatic activity as measured by the ability of lysates from these cells to cleave the synthetic GzB substrate, Ac-IEPD-pNA (Figure 3A). Moreover, in Karpas 299 cell lysates the reduction in Ac-IEPD-pNA cleavage roughly correlated with the degree of GzB knock-down (compare Figures 1 and 3A). Lysates from cells where GzB was knocked-down also had reduced ability to cleave the GzB substrate, vitronectin [5] (Figure 3B).

### GzB sensitizes ALK+ ALCL cell lines to staurosporine-induced apoptosis

**Figure 4:**
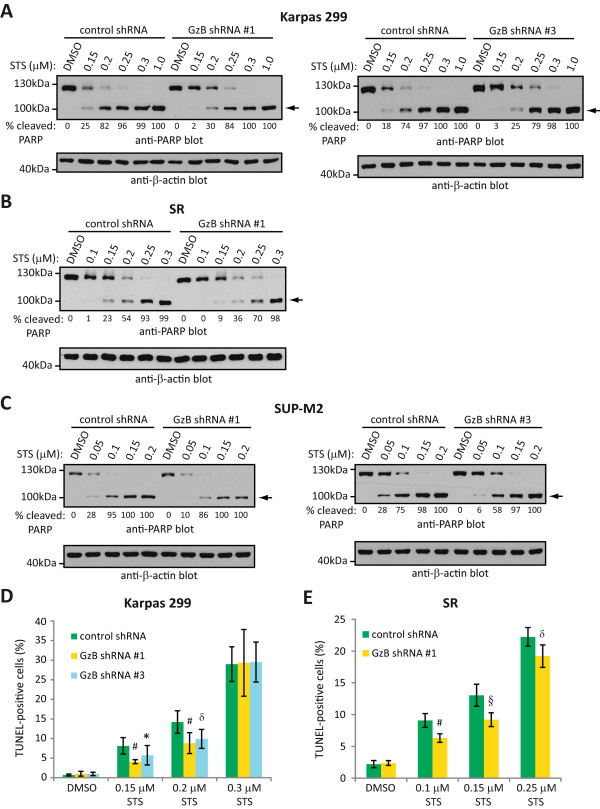
**GzB knock-down reduces the sensitivity of ALK+ ALCL cell lines to staurosporine-induced apoptosis.** Karpas 299 **(A)**, SR **(B)**, or SUP-M2 **(C)** cells expressing either control or GzB shRNA were left untreated (DMSO) or were treated with the indicated concentrations of staurosporine (STS) for 6 h at 37°C. Lysates were then prepared and western blotted with an anti-PARP antibody. The arrow indicates cleaved PARP. The anti-β-actin blot demonstrates equivalent protein loading. The percentage of cleaved PARP (% cleaved PARP) was determined by densitometry and represents the percentage of cleaved PARP as a fraction of total PARP. Molecular mass standards are indicated to the left of the western blots. Karpas 299 **(D)** or SR **(E)** cells expressing either control or GzB shRNA were left untreated (DMSO) or were treated with the indicated concentrations of staurosporine (STS) for 6 h at 37°C. DNA fragmentation was then examined by TUNEL staining and results were expressed as the percentage of TUNEL-positive cells. The results shown represent the mean and standard deviation of at least 4 (Karpas 299) or 5 (SR) independent experiments. *p* values comparing cells expressing GzB shRNA to cells expressing control shRNA were obtained by performing paired, one-tailed t-tests. *p < 0.05, § *p* < 0.01, # *p* < 0.005, δ *p* < 0.001.

GzB expression has been argued to contribute to apoptosis induction in nasal-type NK/T cell lymphoma [12] and prostate cancer cells [18]. Therefore, we investigated whether the expression of GzB in ALK+ ALCL cells might sensitize these cells to spontaneous or drug-induced apoptosis. As a measure of apoptosis, we first examined cleavage of PARP which is a substrate of executioner caspases in apoptotic cells [31–33]. While we observed no difference in PARP cleavage between lysates of DMSO-treated Karpas 299 cells expressing control or GzB shRNAs, at low doses of staurosporine treatment (particularly at 0.15 and 0.2 μM) PARP cleavage was less prominent in lysates from cells with GzB knocked-down compared to cells expressing control shRNA (Figure 4A). A similar reduction in PARP cleavage at low doses of staurosporine treatment was observed in lysates of SR (Figure 4B) and SUP-M2 (Figure 4C) cells expressing GzB shRNA; albeit the difference in PARP cleavage between GzB and control shRNA-expressing cells in the SUP-M2 line was less pronounced. At higher staurosporine concentrations, PARP cleavage was comparable in GzB and control shRNA-expressing cells.

To confirm our findings that GzB knock-down reduces the sensitivity of ALK+ ALCL cell lines to staurosporine-induced apoptosis and to better quantify this difference, we examined the effect of GzB knock-down on the degree of terminal deoxynucleotidyl transferase dUTP nick end labelling (TUNEL)-positive staining in ALK+ ALCL cell lines treated with staurosporine. TUNEL staining measures the characteristic DNA fragmentation of apoptotic cells [34, 35]. Similar to our PARP cleavage data, we found that GzB knock-down had no effect on TUNEL-positive staining in untreated Karpas 299 cells (Figure 4D); however, at lower concentrations of staurosporine treatment (0.15 and 0.2 μM), we observed a 30-50% reduction in the percentage of TUNEL-positive cells in the GzB shRNA-expressing cells compared to the control shRNA-expressing cells (Figure 4D). A ~30% decrease in the percentage of TUNEL-positive cells was also observed in SR cells at lower doses of staurosporine treatment (0.1 and 0.15 μM) when GzB was knocked-down (Figure 4E). We even noted a modest decrease (~14%) in the percentage of TUNEL-positive cells in SR cells with reduced GzB levels when treated with the highest staurosporine concentration (0.25 μM) tested. Taken together, these results demonstrate that GzB sensitizes ALK+ ALCL cell lines to staurosporine-induced apoptosis; however, at higher staurosporine concentrations GzB expression does not appear to significantly contribute to apoptosis induction.

### GzB sensitizes the SUP-M2 and SR ALK+ ALCL cell lines to doxorubicin-induced PARP cleavage

**Figure 5:**
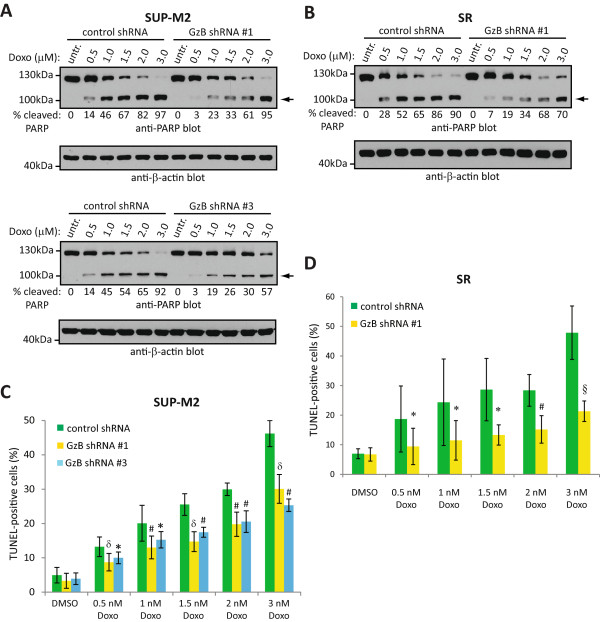
**GzB knock-down reduces the sensitivity of ALK+ ALCL cell lines to doxorubicin-induced apoptosis.** SUP-M2 **(A)** or SR **(B)** cells expressing either control or GzB targeting shRNA were left untreated (untr.) or were treated with indicated concentrations of doxorubicin (Doxo) for 12 h at 37°C. Cells were then lysed and lysates were probed with an anti-PARP antibody. The arrow indicates cleaved PARP. The anti-β-actin blot demonstrates equivalent protein loading. The percent cleaved PARP (% cleaved PARP) was determined by densitometry and represents the percentage of cleaved PARP as a fraction of total PARP. Molecular mass standards are indicated to the left of the western blots. SUP-M2 **(C)** or SR **(D)** cells expressing either control or GzB shRNA were left untreated (DMSO) or were treated with the indicated concentrations of doxorubicin (doxo) for 12 h at 37°C. DNA fragmentation was then examined by TUNEL staining and results were expressed as the percentage of TUNEL-positive cells. The results shown represent the mean and standard deviation of 4 independent experiments. *p* values comparing cells expressing GzB shRNA to cells expressing control shRNA were obtained by performing paired, one-tailed t-tests. *p < 0.05, § *p* < 0.01, # *p* < 0.005, δ *p* < 0.001.

Doxorubicin is a DNA damaging agent that induces apoptosis in ALK+ ALCL cell lines [36], and is one component of the CHOP (cyclophosphamide, hydroxydaunorubicin (doxorubicin), oncovin, and prednisone) chemotherapy regimen often used to treat ALK+ ALCL patients [37–39]. Therefore, we investigated whether GzB also sensitized ALK+ ALCL cells to doxorubicin-induced apoptosis. In both SUP-M2 (Figure 5A) and SR (Figure 5B) cells, we observed reduced PARP cleavage in response to doxorubicin treatment in the GzB shRNA-expressing cells compared to control shRNA-expressing cells. Moreover, we also observed a decrease in the percentage of TUNEL positive cells in GzB knockdown SUP-M2 (Figure 5C) and SR (Figure 5D) cells. Thus, the increased sensitivity to drug-induced apoptosis conferred by GzB expression is common to multiple drugs.

### Over-expression of GzB increases the sensitivity of the Karpas 299 cell line to staurosporine-induced apoptosis

**Figure 6:**
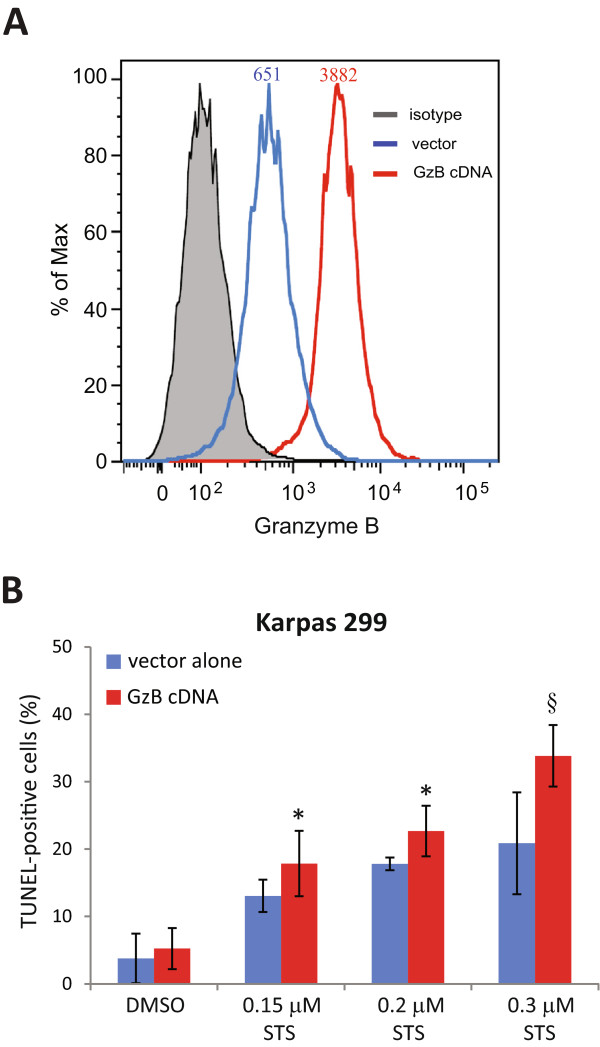
**GzB over-expression increases the sensitivity of Karpas 299 cells to staurosporine-induced apoptosis. A**. Representative flow cytometry plot showing GzB expression in Karpas 299 cells transfected with either a GzB cDNA (red line) or vector alone blue line). The shaded histogram indicates cells stained with an irrelevant isotype control antibody. The numbers above each peak represent the mean fluorescence intensity of GzB staining in vector and GzB cDNA transfected cells. **B**. Cells in **A**. were treated for 6 h with staurosporine at the indicated concentrations and apoptosis was measured by TUNEL. The results represent the average and standard deviation of 5 independent experiments. *p* values comparing cells transfected with GzB cDNA to cells transfected with vector alone were obtained by performing paired, one-tailed t-tests. *p < 0.05, § *p* < 0.01.

Since knock-down of GzB reduced the sensitivity of ALK+ ALCL cell lines to drug-induced apoptosis, we examined whether GzB over-expression might further sensitize these cells to drug induced apoptosis. We chose the Karpas 299 cell line because this line possesses the least amount of endogenous GzB, and in our hands, is the most efficiently transfected ALK+ ALCL cell line (results not shown). Transfection of Karpas 299 cells with a GzB cDNA resulted in a ~4-8-fold increase in GzB expression (Figure 6A). Using TUNEL to measure apoptosis induction, we found that GzB-transfected Karpas 299 cells were modestly more sensitive to staurosporine treatment compared to cells transfected with vector alone (Figure 6B). These findings further implicate GzB expression as a sensitizing factor to drug-induced apoptosis in ALK+ ALCL cell lines.

### GzB is the only granzyme expressed at significant levels in ALK+ ALCL cell lines

**Figure 7:**
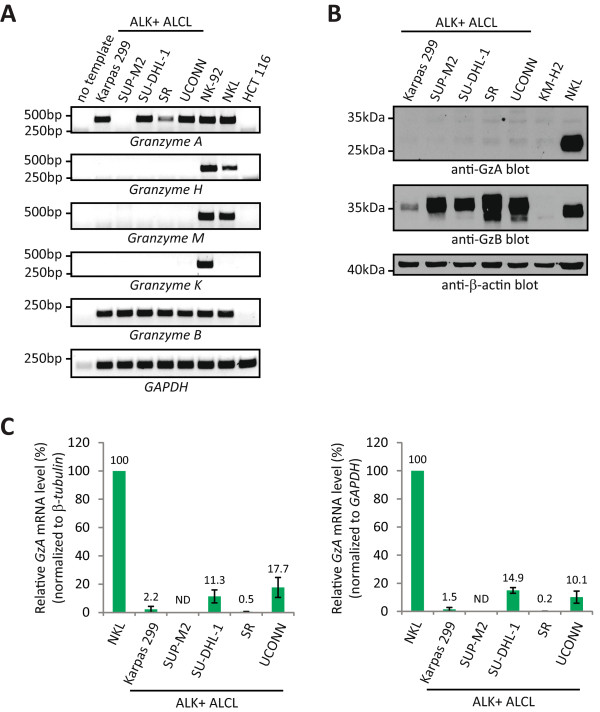
**Expression of granzymes in ALK+ ALCL cell lines. A**. RT-PCR was performed on RNA isolated from the indicated ALK+ ALCL cell lines to examine the expression of the indicated genes. RNA isolated from the NKL (human NK leukemia) and NK-92 (human NK lymphoma) cells served as positive controls for granzyme expression, whereas RNA isolated from HCT116 (human colon carcinoma) cells served as a negative control. In the “no template” sample, no template cDNA was used in the PCR reaction. Base pair standards are indicated to the left of images. **B**. Western blot analysis examining GzA and GzB expression in the lysates of ALK+ ALCL cell lines. The KM-H2 cell line (Hodgkin lymphoma) serves as a negative control, while the NKL cell line serves as a positive control for GzA protein expression. Molecular mass standards are indicated to the left of the western blots. **C**. Quantitative RT-PCR was performed to compare *GzA* mRNA expression levels between the ALK+ ALCL cell lines and the NKL cell line. The results were normalized to either *β-tubulin* (left) or *GAPDH* (right) levels. The results represent the average and standard deviation of three independent experiments. ND = not detected.

There are five human granzymes (A, B, H, K, and M) and each of these can induce cell death when added as a purified protein to cells along with perforin or another agent to deliver the granzyme into the cytosol of the target cell [40–47]. While data demonstrating the importance of some of these granzymes in mediating cell death *in vivo* is lacking, GzA has been implicated in the killing of target cells by CTL and NK cells via a mechanism that shares many characteristics with GzB-induced apoptosis [48–50]. Furthermore, NK cells can use GzK to kill activated T cells [51]. Therefore, we investigated whether other human granzymes might also sensitize ALK+ ALCL cell lines to cell death. Since, to our knowledge, the expression of the granzymes A, H, K, and M has not been described in this lymphoma, we performed reverse transcriptase-polymerase chain reaction (RT-PCR) to examine whether they are expressed in ALK+ ALCL cell lines. The NK cell lines, NKL and NK-92, express multiple human granzymes and were used as positive controls [52, 53], whereas the HCT 116 colorectal carcinoma cell line was used as a negative control for granzyme expression. RT-PCR analysis of RNA collected from these cell lines demonstrated that four of the five ALK+ ALCL cell lines (Karpas 299, SU-DHL-1, SR and UCONN) expressed detectable levels of *GzA* mRNA (Figure 7A). In contrast, we did not detect *GzH*, *GzK* or *GzM* mRNA in any of the ALK+ ALCL cell lines, but the expression of these genes was readily detected in either the NK-92 or NKL cell lines (Figure 7A). *GzB* expression was observed in all the ALK+ ALCL cell lines.

We next performed western blotting experiments to examine the expression of GzA protein in ALK+ ALCL cell lines. Surprisingly, while we could detect GzA protein in NKL cell lysates, we did not observe GzA protein expression in lysates from any of the ALK+ ALCL cell lines (Figure 7B). As expected, GzB protein was detected in all the ALK+ ALCL cell lines. To resolve this discrepancy between our ability to detect GzA expression at the mRNA but not the protein level, we compared *GzA* mRNA levels between the ALK+ ALCL cell lines and NKL cells using quantitative real time-polymerase chain reaction (qRT-PCR). Because the different cell lines could express varying levels of individual housekeeping genes, we normalized our data to both *GAPDH* and *β-tubulin*. Our results showed that the ALK+ ALCL cell lines expressed substantially less *GzA* mRNA than NKL cells, with similar results obtained when *GzA* levels were normalized to each housekeeping gene (Figure 7C). This finding suggests that our inability to detect GzA protein in the ALK+ ALCL cell lines is likely due to the low *GzA* transcript levels in these cells. In sum, the results of these experiments demonstrate that GzB is the only granzyme expressed at significant levels in these ALK+ ALCL cell lines and therefore, is the only granzyme that could be sensitizing these cells to programmed cell death.

## Discussion

In this report, we provide evidence linking the expression of GzB to apoptosis sensitivity in ALK+ ALCL. We show that knock-down of GzB in multiple ALK+ ALCL cell lines using several distinct GzB shRNAs resulted in reduced apoptosis following treatment with low doses of staurosporine. This was demonstrated by a reduction in both PARP cleavage and the percentage of TUNEL-positive cells (Figure 4). Moreover, we observed increased staurosporine-induced apoptosis in Karpas 299 cells over-expressing GzB (Figure 6). We also observed a similar impairment in PARP cleavage and reduced TUNEL staining in SUP-M2 and SR cells lines expressing GzB shRNA following treatment with doxorubicin (Figure 5), a component of the combination chemotherapy used to treat patients with ALK+ ALCL [37–39]. Surprisingly, we observed no impairment in PARP cleavage, and even a slight enhancement in some experiments, when Karpas 299 cells expressing GzB shRNA were treated with doxorubicin (unpublished observation). Why this is the case is unclear since we observed a clear effect on doxorubicin-induced PARP cleavage with GzB knock-down in other ALK+ ALCL cell lines (Figure 5), and found that knocking-down GzB expression in Karpas 299 cells reduced the sensitivity of these cells to staurosporine-induced apoptosis (Figure 4A and D). Independent of this one inconsistent result, our findings strongly implicate GzB expression in ALK+ ALCL as a sensitizing factor to apoptosis-inducing drugs.

An unresolved question from this study is how GzB sensitizes ALK+ ALCL cell lines to drug-induced apoptosis. In nasal NK/T cell lymphomas GzB has been argued to spontaneously “leak” from cytoplasmic granules and initiate apoptosis [12]. However, since we observed no decrease in apoptosis associated with GzB knock-down in untreated cells (Figures 4 and 5), it is unlikely that spontaneous leakage of GzB is contributing to apoptosis in ALK+ ALCL cell lines. Rather, we favour a model whereby drug treatment initiates apoptosis in cells resulting in damage to the cytotoxic vesicles and release of GzB into the cytosol. This phenomenon has been observed in CD8+ CTLs induced to undergo apoptosis by treatment with the human cathelicidin, LL-37 [54]. Once in the cytosol, we postulate that GzB then amplifies apoptosis induction. The release of GzB into the cytosol would be predicted to be particularly detrimental in ALK+ ALCL, as tumour cells in ALK+ ALCL patient samples do not express the GzB inhibitor, proteinase inhibitor-9 (PI-9) [28]. PI-9 is an irreversible inhibitor of GzB and it is expressed by cytotoxic cells as a means of protecting these cells from GzB that enters the cytoplasm [55]. It is also expressed in some tumour cells and postulated to be a means by which these cells protect themselves from being killed by CTLs and NK cells [55]. We have found that PI-9 is either not expressed or expressed at very low levels in ALK+ ALCL cell lines (unpublished observation), and we suspect this is a factor contributing to why GzB sensitizes these cells to apoptosis.

ALK+ ALCL patients are usually treated successfully using standard chemotherapy regimens, and the overall 5-year survival rate of these patients is 70-85% [37, 38, 56–58]. One key factor likely impacting favourable prognosis in this lymphoma is the relatively young age (average age of 16–22) of ALK+ ALCL patients [37, 58, 59]. It is probable that ALK+ ALCL tumours have not acquired many secondary oncogenic mutations/alterations that would be predicted to render these tumour cells less susceptible to chemotherapy. Rather, ALK+ ALCL tumour cells are heavily reliant on signalling pathways activated by ALK fusion proteins, and ALK+ status is a strong favourable prognostic indicator in ALCL [37, 38, 56–58, 60]. Our results suggest that the expression of GzB, combined with a lack of PI-9 expression, may be another factor contributing to the favourable clinical outcome of ALK+ ALCL patients.

Interestingly, a previous study found that cytotoxic phenotype, as measured by expression of GzB and TIA-1, did not correlate with clinical outcome in ALCL patients [9]. However, this study included only 13 ALK+ ALCL patients; almost all which possessed a cytotoxic phenotype (12 of 13) and all had favourable outcome. Thus, we feel that a more extensive study investigating whether cytotoxic phenotype, and more specifically expression of GzB, is important in clinical outcome is warranted. Moreover, since the number of GzB-positive cells vary quite significantly in ALK+ ALCL patients [8–10], it will be important to examine whether the degree of GzB expression is clinically relevant in this lymphoma. Regardless of whether or not GzB expression is prognostic or predictive of ALK+ ALCL outcome, our results clearly demonstrate that it is a factor contributing to the susceptibility of these tumour cells to drug-induced apoptosis.

## Conclusions

Our findings reveal that GzB expression is more than just a phenotypic characteristic of ALK+ ALCL lymphoma cells, as expression of this serine protease sensitizes ALK+ ALCL tumour cells to drug-induced apoptosis. Furthermore, we postulate that this sensitization may be one factor contributing to the successful treatment and the favourable outcome of ALK+ ALCL patients.

## Methods

### Antibodies and other reagents

The mouse monoclonal antibodies against Granzyme B (2C5), β-actin (AC-15), and PARP (C2-10), as well as the vitronectin polyclonal antibody (H-270) were obtained from Santa Cruz Biotechnology (Santa Cruz, CA). The polyclonal antibody to Granzyme A (#4928) was purchased from Cell Signaling Technology (Danvers, MA). Purified human vitronectin was obtained from BD Biosciences (Mississauga, ON) and the synthetic GzB substrate Ac-IEPD-pNA was purchased from Sigma-Aldrich (St. Louis, MO). Staurosporine was obtained from Enzo Life Sciences (Plymouth Meeting, PA) and doxorubicin was purchased from Sigma-Aldrich.

### Cell lines

The Karpas 299 and SUP-M2 ALK+ ALCL cell lines were purchased from the Leibniz Institute DSMZ-German Collection of Microorganisms and Cell Cultures (Braunschweig, Germany). The ALK+ ALCL cell line SR (also known as SR-786) was obtained from the American Type Culture Collection (Manassas, VA) and the SU-DHL-1 and UCONN ALK+ ALCL cell lines were provided by Dr. Raymond Lai (University of Alberta, Edmonton, AB, Canada). The Hodgkin lymphoma cell line, KM-H2, was generously provided by Dr. Hesham Amin (University of Texas M. D. Anderson Cancer Center, Houston, TX). KM-H2 and ALK+ ALCL cell lines were cultured in RPMI 1640 media supplemented with 10% heat-inactivated fetal bovine serum (FBS), 1 mM sodium pyruvate (Sigma-Aldrich; St Louis, MO), 2 mM L-glutamine (Gibco; Burlington, ON, Canada), and 50 μM 2-mercaptoethanol (BioShop; Burlington, ON, Canada). The human leukemic NK cell line, NKL, and the non-Hodgkin lymphoma NK cell line, NK-92, were generously provided by Dr. Debby Burshtyn (University of Alberta). NKL cells were cultured in Iscove’s media supplemented with 2 mM L-glutamine, 10% FBS and 200 U/ml recombinant human interleukin (IL)-2 (Sigma-Aldrich). NK-92 cells were cultured in Iscove’s media supplemented with 2 mM L-glutamine, 12.5% FBS, 12.5% horse serum, 36 μg/ml inositol (Sigma Aldrich), 17.6 μg/ml folic acid (Sigma-Aldrich), 100 μM 2-mercaptoethanol, and 100 U/ml recombinant human IL-2. The HCT 116 colorectal carcinoma cell line (a gift from Dr. David Murray; University of Alberta) and HEK 293T cells were cultured in DMEM supplemented with 10% FBS, 1 mM sodium pyruvate and 2 mM L-glutamine. All cells were maintained at 37°C in a 5% CO_2_ atmosphere.

### Generating stable ALK+ ALCL cell lines using lentiviral transduction

HEK 293T cells were used to generate lentiviral vectors using the MISSION short hairpin RNA (shRNA) lentiviral system (Sigma-Aldrich). HEK 293T cells were transfected with lentiviral packaging vectors along with pLKO.1 vector containing the shRNA sequence of interest using the FuGENE HD transfection reagent (Promega; Madison, WI). The shRNA constructs used were: control, non-targeting shRNA (SHC002) and three different GzB shRNA constructs designated #1 (TRCN0000006445), #2 (TRCN0000006448) and #3 (TRCN0000006449). Approximately 48 h after transfection, lentivirus-containing supernatant was collected from the transfected HEK 293T cells and used to infect the ALK+ ALCL cell lines. Pooled populations of stable transfectants were then selected in 0.5 μg/ml puromycin and GzB knock-down was assessed by western blotting and flow cytometry.

### Staurosporine and doxorubicin treatments

To induce apoptosis in ALK+ ALCL cell lines, cells were counted and resuspended at 4–6x10^5^ cells/ml in complete RPMI media. Cells were then treated with 0.1% DMSO (untreated) or the indicated concentrations of staurosporine for 6 h at 37°C. Alternatively, cells were left untreated or were treated with the indicated concentrations of doxorubicin for 12 h at 37°C.

### Cell lysis and western blotting

Cells were collected by centrifugation and lysed in Nonidet P-40 lysis buffer [61] containing protease inhibitor cocktail (Sigma-Aldrich; Mississauga, ON, Canada), 1 mM phenylmethylsulfonylfluoride, and 1 mM sodium orthovanadate. Lysates were then cleared of detergent-insoluble material by centrifugation for 10 minutes at ~20,000 *g*, and the protein concentration of lysates was determined using the BCA Protein Assay kit (Thermo Scientific; Waltham, MA). For western blots, equivalent protein amounts were resolved on SDS-PAGE gels, transferred to nitrocellulose membranes, probed with the indicated antibodies and then visualized using SuperSignal West Pico Chemiluminescent Substrate (Thermo Scientific). Reprobed blots were first stripped in 0.1% TBST, pH 2 and then probed with the new primary antibody. The amount of cleaved PARP or vitronectin was quantified by densitometry using ImageJ software (NIH, Bethesda, MD).

### Analysis of GzB expression using flow cytometry

Cells (~4x10^5^) were collected by centrifugation and permeabilized using BD Cytofix/Cytoperm solution (BD Biosciences; Mississauga, ON, Canada) for 20 min on ice. Cells were washed once in BD Perm/Wash buffer (BD Biosciences) and resuspended in BD Perm/Wash buffer containing 1:250 diluted APC-conjugated anti-GzB (clone GB12) or isotype control antibody (Invitrogen; Burlington, ON, Canada). Cells were incubated with the antibody for 30 min on ice, washed once with BD Perm/Wash buffer and analysed by flow cytometry using a BD FACSCalibur (Figure 1) or a BD LSR Fortessa (Figure 6) flow cytometer (BD Biosciences). Data were analysed using CellQuest Pro (BD Biosciences), FACSDiva (BD Biosciences), and FlowJo software (Tree Star Inc.; Ashland, OR).

### Measuring the growth rate of cell lines

Cells (5x10^4^/ml) were plated in fresh media and cell viability was measured in triplicate each day using a Resazurin-based assay [62] on a FLUOstar OPTIMA microplate reader (BMG Labtech; Ortenberg, Germany).

### Cleavage of the synthetic GzB substrate, Ac-IEPD-pNA

The level of GzB activity present in ALK+ ALCL cell lysates was assessed by measuring cleavage of the synthetic GzB substrate, Ac-IEPD-pNA, as previously described [63]. Cells were lysed at a concentration of 5x10^7^ cells/ml as outlined above, with the exception that protease inhibitors were omitted from the lysis buffer. Lysates were then incubated with 200 μM Ac-IEPD-pNA at 37°C for 30 min and absorbance was measured at 405 nm using a BMG Labtech FLUOstar OPTIMA microplate reader (Ortenberg, Germany). Each sample was assayed in triplicate and the triplicate measurements were averaged. Results are presented relative to the activity present in lysate from cells expressing control shRNA, which was set to 100%.

### Vitronectin cleavage assays

Wells of a 96-well plate were coated with 0.5 μg of purified human vitronectin (suspended in 50 μl of 1X PBS) at 4°C overnight. Wells were then blocked with 2% BSA (in 50 μl of 1X PBS) for 1 hr at 37°C, and washed twice with 1X PBS. The indicated cell lines were then lysed at 5x10^7^ cells/ml in lysis buffer lacking protease inhibitors, and 50 μl of cell lysate was added to the coated wells. Plates were then incubated at 37°C for the indicated times, at which time the reactions were stopped by the addition of SDS-PAGE sample buffer. Samples were resolved using SDS-PAGE and vitronectin cleavage was examined by western blotting. The amount of cleaved vitronectin was quantified by densitometry using ImageJ software.

### TUNEL

Apoptosis was assayed in staurosporine-treated cells using TUNEL with the *In Situ* Cell Death Detection Kit, Fluorescein (Roche Applied Science; Laval, QC, Canada) as outlined in the manufacture’s protocol. Following TUNEL staining, cells were analysed using a FACSCalibur or a BD LSR Fortessa flow cytometer to determine the percentage of dUTP-positive cells.

### Transfection of Karpas 299 cells

1x10^7^ Karpas 299 cells were transfected with 50 μg of GzB cDNA in pcDNA 3.1A (Invitrogen) or 50 μg of pcDNA 3.1A alone as previously described [64] using a BTX square wave electroporator (Harvard Apparatus; Holliston MA). 48 h post-transfection, cells were treated with staurosporine and apoptosis was analysed by TUNEL as described above.

### RNA extraction and RT-PCR analysis

Total RNA was collected from the indicated cell lines using the RNeasy mini kit (Qiagen; Mississauga, ON). The RNA was then digested with DNase I, and converted to cDNA using the Superscript II Reverse Transcriptase System (Invitrogen). The cDNA that was generated was then subjected to PCR using the following primer sets: *GzB* forward - TGC GAA TCT GAC TTA CGC CAT, reverse - GGA GGC ATG CCA TTG TTT CG; *GzA* forward - CAT CTG TGC TGG GGC TTT GA, reverse - GAG GCT TCC AGC ACA AAC CA; *GzH* forward - GCA AGA GAA GAG TCG GAA GAG G, reverse - AAC CCC AGC CAG CCA CAC; *GzK* forward - CGT TTG TGG AGG TGT TCT GAT TG, reverse - CAG TGA CTT CTC GCA GGG TG; *GzM* forward - GCG GGG GTG TCC TGG TG, reverse - ATG CTG GGG GAG AGG CTG; and *GAPDH* forward - GAC AGT CAG CCG CAT CTT CT, reverse - TTA AAA GCA GCC CTG GTG AC. PCR products were resolved using agarose gel electrophoresis and visualized by staining with SYBR Safe DNA gel stain (Invitrogen).

### qRT-PCR analysis

RNA was collected and converted to cDNA as described above. qRT-PCR was performed using PerfeCTa SYBR Green FastMix (Quanta Biosciences; Gaithersburg, MD) on a Bio-Rad CFX96 Real-Time PCR Detection System (Bio-Rad). *GzA* mRNA levels were then determined using the ΔΔ-CT method [65] with *β-tubulin* or *GAPDH* as the housekeeping genes. The following primer sets were used: *GzA* forward - CAT CTG TGC TGG GGC TTT GA, reverse - TCT GTT TTG TTG GCT CTT CCC T; *GAPDH* forward - GAC AGT CAG CCG CAT CTT CT, reverse - TTA AAA GCA GCC CTG GTG AC; and *β-tubulin* forward - CAG GCT GGT CAG TGT GGC A, reverse - CAG GAT GGC ACG AGG AAC.

## Abbreviations

ALK+ ALCL: Anaplastic lymphoma kinase-positive anaplastic large cell lymphoma
CHOP: Cyclophosphamide, hydroxydaunorubicin (doxorubicin), oncovin, and prednisone
CTL: Cytotoxic T lymphocyte
ECM: Extracellular matrix
FBS: Fetal bovine serum
GzB: Granzyme B
ICAD: Inhibitor of caspase-activated DNase
IL-2: Interleukin-2
NK: Natural killer
PARP: Poly (ADP-ribose) polymerase
PI-9: Proteinase inhibitor-9
qRT-PCR: Quantitative real-time polymerase chain reaction
RT-PCR: Reverse transcriptase-polymerase chain reaction
shRNA: Short-hairpin RNA
TIA-1: T cell-restricted intracellular antigen-1
TUNEL: Terminal deoxynucleotidyl transferase dUTP nick end labelling.
