# Erratum to: ‘Expression of granzyme B sensitizes ALK+ ALCL tumour cells to apoptosis-inducing drugs’

**DOI:** 10.1186/s12943-016-0499-1

**Published:** 2016-03-01

**Authors:** Jodel D. Pearson, Jingxi Zhang, Zuoqiao Wu, Kayla D. Thew, Katelynn J. Rowe, Julinor T. C. Bacani, Robert J. Ingham

**Affiliations:** Department of Medical Microbiology and Immunology and Li Ka Shing Institute of Virology, University of Alberta, Katz Group Centre for Pharmacy and Health Research, University of Alberta, Edmonton, Canada; Department of Laboratory Medicine and Pathology, University of Alberta, Edmonton, Canada

Unfortunately, the original version of this article [1] contained an error. A figure was mislabelled. In Figs. 5c and d the Doxorubicin (Doxo) concentrations should be in μM. Here is most recent version of the figure with it correctly labelled.

**Fig. 5:**
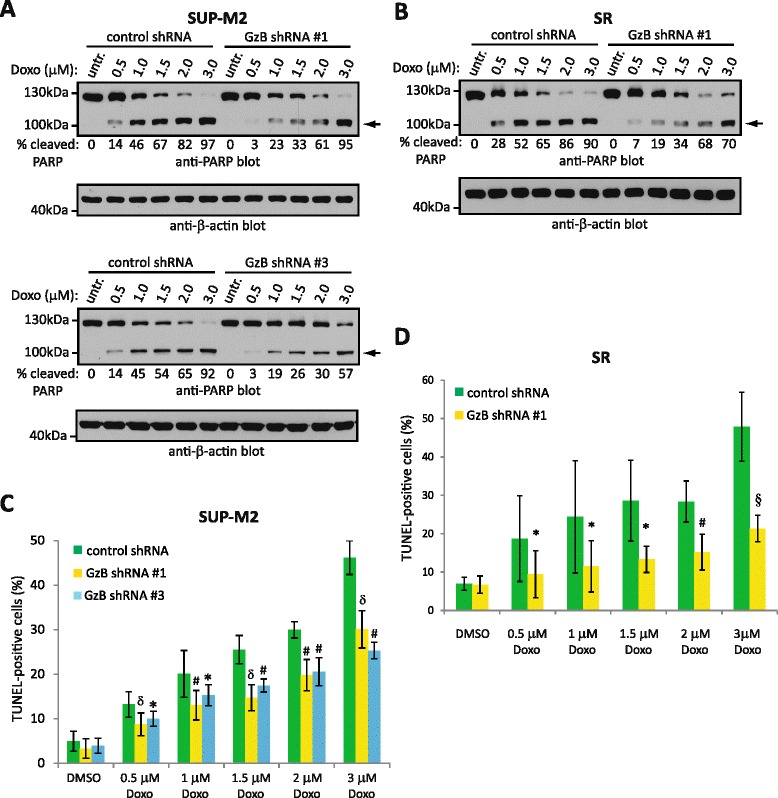
From: Expression of granzyme B sensitizes ALK + ALCL tumour cells to apoptosis-inducing drugs. GzB knock-down reduces the sensitivity of ALK + ALCL cell lines to doxorubicin-induced apoptosis. SUP-M2 (**a**) or SR (**b**) cells expressing either control or GzB targeting shRNA were left untreated (untr.) or were treated with indicated concentrations of doxorubicin (Doxo) for 12 h at 37 °C. Cells were then lysed and lysates were probed with an anti-PARP antibody. The arrow indicates cleaved PARP. The anti-β-actin blot demonstrates equivalent protein loading. The percent cleaved PARP (% cleaved PARP) was determined by densitometry and represents the percentage of cleaved PARP as a fraction of total PARP. Molecular mass standards are indicated to the left of the western blots. SUP-M2 (**c**) or SR (**d**) cells expressing either control or GzB shRNA were left untreated (DMSO) or were treated with the indicated concentrations of doxorubicin (doxo) for 12 h at 37 °C. DNA fragmentation was then examined by TUNEL staining and results were expressed as the percentage of TUNEL-positive cells. The results shown represent the mean and standard deviation of 4 independent experiments. p values comparing cells expressing GzB shRNA to cells expressing control shRNA were obtained by performing paired, one-tailed t-tests. *p < 0.05, §*p* < 0.01, #*p* < 0.005, δ*p* < 0.001
